# One fold, two functions: cytochrome P460 and cytochrome *c*′-β from the methanotroph *Methylococcus capsulatus* (Bath)†
†Electronic supplementary information (ESI) available. See DOI: 10.1039/c8sc05210g


**DOI:** 10.1039/c8sc05210g

**Published:** 2019-01-21

**Authors:** Hannah R. Adams, Callie Krewson, Jenny E. Vardanega, Sotaro Fujii, Tadeo Moreno, Chicano, Yoshihiro Sambongi, Dimitri Svistunenko, Jordi Paps, Colin R. Andrew, Michael A. Hough

**Affiliations:** a School of Biological Sciences , University of Essex , Wivenhoe Park , Colchester , Essex CO4 3SQ , UK . Email: mahough@essex.ac.uk; b Department of Chemistry and Biochemistry , Eastern Oregon University , La Grande , Oregon 97850 , USA . Email: candrew@eou.edu; c Graduate School of Biosphere Science , Hiroshima University , Kagamiyama 1-4-4, Higashi-Hiroshima , Hiroshima , 739-8528 , Japan

## Abstract

Nature is adept at utilising highly similar protein folds to carry out very different functions, yet the mechanisms by which this functional divergence occurs remain poorly characterised. In certain methanotrophic bacteria, two homologous pentacoordinate c-type heme proteins have been identified: a cytochrome P460 (cyt P460) and a cytochrome *c*′-β (cyt cp-β). Cytochromes P460 are able to convert hydroxylamine to nitrous oxide (N_2_O), a potent greenhouse gas. This reactivity is similar to that of hydroxylamine oxidoreductase (HAO), which is a key enzyme in nitrifying and methanotrophic bacteria. Cyt P460 and HAO both have unusual protein-heme cross-links, formed by a Tyr residue in HAO and a Lys in cyt P460. In contrast, cyts cp-β (the only known cytochromes *c*′ with a β-sheet fold) lack this crosslink and appears to be optimized for binding non-polar molecules (including NO and CO) without enzymatic conversion. Our bioinformatics analysis supports the proposal that cyt cp-β may have evolved from cyt P460 *via* a gene duplication event. Using high-resolution X-ray crystallography, UV-visible absorption, electron paramagnetic resonance (EPR) and resonance Raman spectroscopy, we have characterized the overall protein folding and active site structures of cyt cp-β and cyt P460 from the obligate methanotroph, *Methylococcus capsulatus* (Bath). These proteins display a similar β-sheet protein fold, together with a pattern of changes to the heme pocket regions and localised tertiary structure that have converted a hydroxylamine oxidizing enzyme into a gas-binding protein. Structural comparisons provide insights relevant to enzyme redesign for synthetic enzymology and engineering of gas sensor proteins. We also show the widespread occurrence of cyts cp-β and characterise their phylogeny.

## Abstract 2

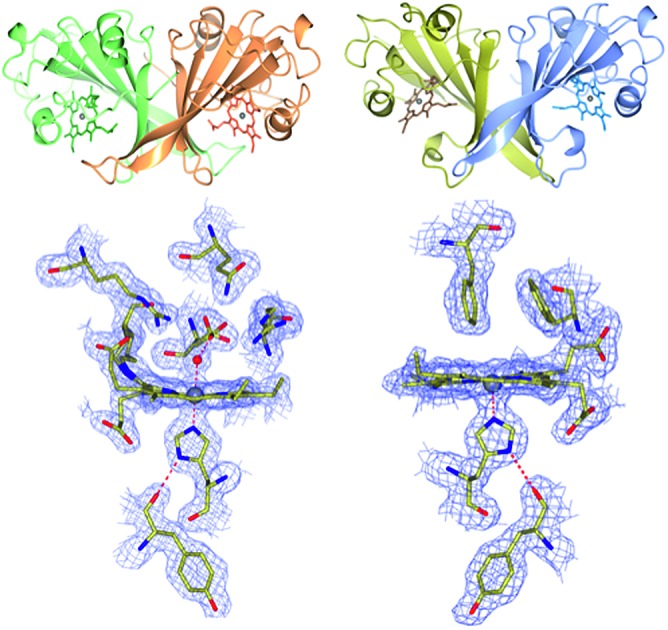
Structural and spectroscopic characterisation of cytochrome *c*′*-*β and cytochrome P460 from *M. capsulatus* (Bath) reveals highly similar protein folds but very different heme pockets, providing functional insights.

## Introduction

Understanding the means by which enzymes can utilise a common fold and structural motifs to evolve very different functions is essential for synthetic biology and rational enzyme design for biotechnology. A useful guide in nature can be found in proteins within the same organism that diverge to adopt different functional roles, typically following a gene duplication event. Methanotrophic bacteria, whether or not they are also nitrifiers, are of considerable interest for biotechnological applications. Methanotrophs are a subgroup of methylotrophic bacteria, which utilise methane as their sole source of carbon and energy^1^ and possess the enzyme methane monooxygenase (MMO) which catalyses the oxidation of methane to methanol.^2^ Some methanotrophs, such as *Methylococcus capsulatus* (Bath), are also an ammonia-oxidising nonlithotrophic bacteria (ANB), organisms that can aerobically oxidise ammonia to nitrite but (unlike nitrifiers) do not use this as their source of energy.^3^ Nitrous oxide is produced by ANB from nitrite reduction (part of nitrifier denitrification) and also hydroxylamine oxidation. In *M. capsulatus* (Bath), two related β-sheet proteins (31% sequence identity^4^) have been previously identified, a cytochrome P460 (McP460) and a cytochrome *c*′ (McCP-β)^5^,^6^ encoded by the *CytL* and *CytS* genes respectively. Cytochromes P460, defined by an unusual porphyrin-Lys cross link at the 13′ meso carbon, catalyse the oxidation of hydroxylamine (NH_2_OH) to form N_2_O under anaerobic conditions. The apparent production of nitrite during this reaction under aerobic conditions has more recently been shown to be due to oxidation of an NO intermediate by oxygen in a non-enzymatic manner.^7^ The hydroxylamine substrate is a toxic intermediate generated as a result of ammonia monooxygenase or methane monooxygenase activity in ammonia oxidising and methane oxidising bacteria (AOB or MOB) respectively.^8^ Hydroxylamine oxidation also occurs in the unrelated hydroxylamine oxidoreductases (HAO) which, in addition to containing multiple heme centers, have a different type of P460 porphyrin cross-link involving a conserved Tyr, as well as very different protein folds.^9^–^11^ The HAO P460 heme pocket also contains Tyr, Asp and His residues implicated in reactivity.^9^–^11^ Structures of cyt P460 12 and HAO^9^,^10^ from the ammonia oxidising bacterium *Nitrosomonas europea*, as well as cyt P460 from *Nitrosomonas* sp. AL212,^13^ have previously been determined, but no structural data have been available for their methanotrophic equivalents. *Nitrosomonas europea* cyt P460 (NeP460) and *Nitrosomonas* sp. AL212 (NsALP460) have been the focus of several recent spectroscopic studies.^12^–^15^ A relationship between heme P460 character, the protein crosslink, and distortion of the heme geometry away from planarity has recently been proposed.^13^

Cytochromes *c*′ (cyts cp) are defined by a pentacoordinate heme Fe with a CXXCH c-heme-binding motif located close to the C-terminus.^16^,^17^ Whereas all cyt cp crystal structures to date have revealed 4 α-helix bundles, circular dichroism studies of McCP-β predicted a predominantly β-sheet fold,^5^ suggesting that there are two distinct types of cytochrome *c*′ proteins, designated cyt cp-α or cyt cp-β according to their secondary structure. Within the cyt cp-α family, reactivity at the distal heme site is governed by a buried and crowded hydrophobic environment that selects for small non-polar ligands (NO and CO) that do not require H-bond stabilization (reviewed in ref. 16)). Moreover, distal steric constraints, coupled with a solvent-exposed proximal heme pocket, result in an unusual proximally-bound five-coordinate heme nitrosyl complex (5cNO or 5c{FeNO}^7^) formed *via* distal six-coordinate heme nitrosyl (6cNO or 6c{FeNO}^7^) and a transient dinitrosyl species.^18^–^20^ Functional roles for cyt cp-α in defence against nitrosoative stress and NO transport have been proposed, while the mechanism of 6cNO → 5cNO conversion is similar to that of the mammalian NO-sensor, soluble guanylate cyclase,^21^ for which no structural data of the haem domain are currently available.

By contrast with cyts cp-α, much less is known about the cyt cp-β family, the sole characterized example to date being that of *M. capsulatus* (Bath) (McCP-β). Data including the amino acid sequence and circular dichroism spectra have been reported for McCP-β,^4^,^5^ together with some ligand binding and spectropotentiometric studies.^6^ These studies suggested that McCP-β is a homodimeric heme protein with a somewhat higher molecular weight (16 kDa per monomer) than cyts cp-α (∼12 kDa per monomer), and with a predicted β-sheet structure. Although McCP-β exhibits some of the ligand binding and spectroscopic properties of cyts cp-α, its reduction potential (–205 mV) is much lower than that of the cyts cp-α (–10 to +202 mV).^22^ A transcriptomics study of *M. capsulatus* (Bath) revealed that exposure to ammonia greatly increased expression (28.5–40-fold) of *CytS* (McCP-β) and of HAO, but not *CytL* (McP460).^23^ This strongly suggests a role for McCP-β in NO scavenging and protection against nitrosoative stress, for example dealing with NO produced by HAO or nitrite reductases in nitrifier denitrification. It has also been suggested that HAO and cyt cp-β are required for N_2_O formation in *M. capsulatus* (Bath)^24^ although upregulation of *CytS* in fact may reflect a protective function against the production of NO by HAO. The lack of *CytL* upregulation in the presence of ammonia may suggest that HAO plays the major role in hydroxylamine oxidation under the conditions used. Interestingly, McCP-β has been shown to act as an electron acceptor from cyt P460 that has been reduced by hydroxylamine, but this is unlikely to be a primary function since cyt cp-β and cyt P460 are not always present in a single organism.^7^ Notably, in NeP460, mutation of the cross-linking Lys to Arg, Ala or Tyr resulted in catalytically inactive proteins with spectroscopic properties similar to those of the cyts cp.^25^

In this study we present high resolution crystal structures of McCP-β and McP460 in their as-isolated states, representing the first crystal structure of any cyt cp-β, and the first for a methanotrophic cyt P460. Despite possessing similar β-sheet protein folds, the heme environment of McCP-β has undergone major structural changes relative to McP460, replacing a highly charged and hydrophilic distal heme pocket with a hydrophobic pocket where two Phe residues act as a ‘cap’ over the distal NO binding position. Characterization of McCP-β and McP460 using UV-visible absorption, EPR and resonance Raman spectroscopies highlights differences in their heme environments, while facilitating comparison with previously characterized proteins from other organisms. Finally, our phylogenetic analysis identifies the occurrence and evolutionary history of these proteins and provides clues to their functional roles. Analysis of these changes in the context of an otherwise highly conserved protein fold provides insights for synthetic enzyme design and synthetic biology.

## Results and discussion

### Crystal structure of McP460 and comparison with other cyts P460

**Fig. 1:**
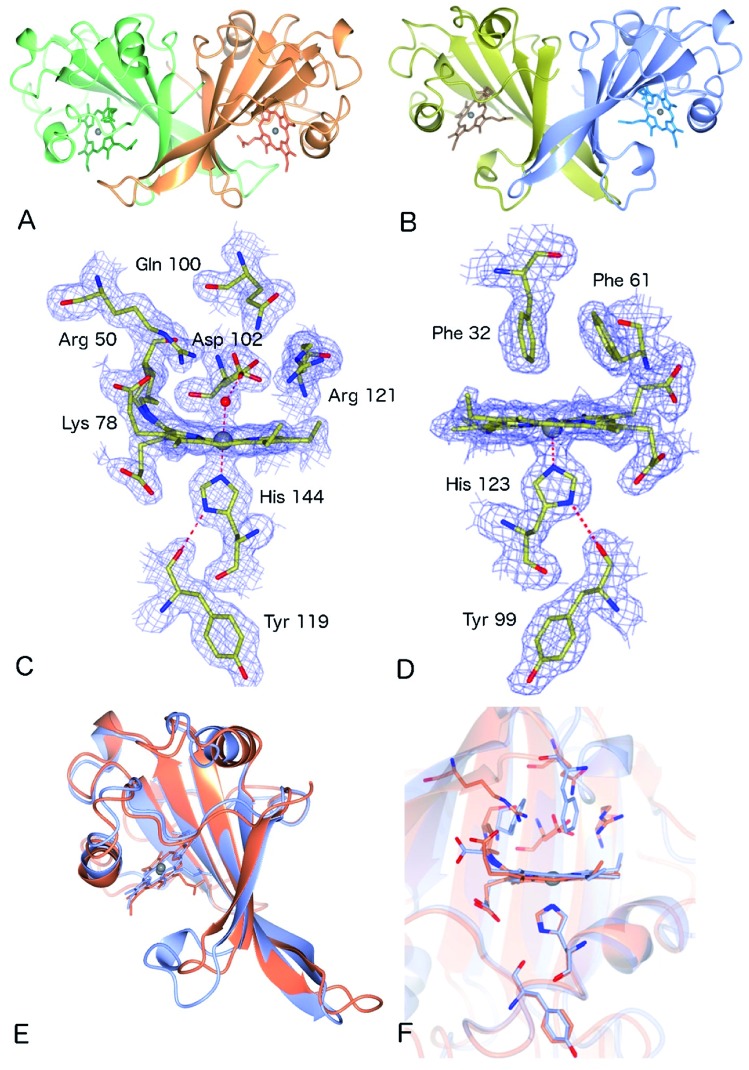
The homodimeric structures of (A) McP460 and (B) McCP-β showing the predominantly β-sheet fold; (C) the heme environment in McP460, note the distal water ligand and hydrophilic, charged pocket with several Arg and Asp residues in a position to interact with bound substrates; (D) the heme environment of McCP-β showing the hydrogen bonding between the proximal His 123 and the backbone carbonyl of Tyr 99. The distal pocket is devoid of water molecules and formed of hydrophobic residues including the ‘Phe Cap’ of Phe 32 and Phe 61; (E) superposition of McCP-β (blue) and McP460 (coral) overall structures; (F) superimposition of the heme environments of McCP-β (blue) and McP460 (coral).

The crystal structure of as-isolated (ferric) McP460 was determined to 1.36 Å resolution (Fig. 1, Table 1) with residues 19 to 161 observed in the electron density maps. McP460 exists as a homodimer, with each monomer consisting of a twisted five-stranded antiparallel β-sheet, two α-helices and two helical turns, Fig. 1A – a protein fold similar to that of NeP460 12 and NsALP460 13 from AOB. The defining P460 covalent cross-link is evident between Lys 78 and the 13′-*meso* carbon of the McP460 porphyrin ring (Fig. 1C). In agreement with a recent study of NsALP460, structural parameters for the *meso* carbon correspond to sp^2^ hybridization, consistent with the retention of porphyrin aromaticity (Table S4†). No hydroxyl modification of the porphyrin (observed at the 5′-*meso* carbon position in NeP460, but not in NsALP460) is evident in our McP460 structure. The proximal pocket of McP460 is also quite similar to that of NeP460 and NsALP460, a minor difference being that the His 144 ligand of McP460 is H-bonded to the Tyr 119 peptide carbonyl, whereas the His ligands of NeP460 and NsALP460 are H-bonded to a Met carbonyl.

**Table 1:** Data collection and processing statistics for the crystal structure of McCP-β and McP460

Dataset	McCP-β	McP460
Resolution (Å)	61.18–1.61	59.0–1.36
Space group	*P*2_1_3	*P*2_1_2_1_2_1_
Unit cell, (Å)	*a* = *b* = *c* = 106.0	*a* = 46.6
		*b* = 81.6
		*c* = 85.3
Unique reflections	52 377	70 585
Completeness (%)	99.9 (99.4)	99.9 (98.5)
Redundancy	7.6 (4.0)	5.4 (4.4)
*R*_merge_	0.047 (0.93)	0.043 (1.048)
*I*/*σ*(*I*)	20.9 (1.4)	13.7 (1.2)
CC_1/2_	0.99	1.00 (0.49)
Wilson B-factor (Å^2^)	24.3	16.0
*R*_work_	0.177	0.197
*R*_free_	0.202	0.224
RMSD bond length (°)	0.015	0.021
RMSD bond angles (Å)	2.15	1.84
Ramachandran favoured (%)	96.1	94.6
PDB accession code	6HIH	6HIU

The distal side of the McP460 heme is easily accessible to solvent as there is a large opening on the side of the protein (Fig. S1†). Within the distal heme pocket are a large number of polar residues (Arg 43, Gln 46, Asn 48, Arg 50, Gln 100, Asp 102 and Arg 121) (Fig. 1C)), with a well-defined water molecule bound at a distance of 2.43 Å (Table 2) to produce a 6-coordinate Fe species, in a manner similar to that of the HAO P460 cofactor.^10^ The distal water ligand in McP460 is 3.1/3.2 Å from residue Asp 102, and we note that a bound hydroxylamine substrate displacing the distal water would likely be in hydrogen-bonding distance to several additional pocket residues such as Arg 43, Arg 50, Asp 102 or Arg 121. Interestingly, the distal heme environment of McP460 exhibits significant differences relative to cyts P460 characterized from AOB. Notably, NsALP460 has a much more hydrophobic distal pocket environment than McP460 (or NeP460), without residues likely to form a stabilising hydrogen bonds to distal ligands, Fig. S2.† Indeed, in certain respects, the NsALP460 structure resembles McCP-β as much as it does NeP460 or McP460 (see below). The NsALP460 heme is 5c with no evidence of a distal water molecule or other ligand,^13^ while in the NeP460 crystal structure,^12^ a phosphate anion from the crystallisation medium obscures the coordination of the Fe(iii) resting state. By contrast, our structure of McP460 clearly shows a 6c Fe(iii) heme resting state, similar to that of the HAO P460 cofactor. The presence of a water ligand in NeP460 remains an open question and awaits determination of a structure for that enzyme in the absence of phosphate.

**Table 2:** Heme site parameters for McCP-β and P460 structures

Structure	McCP-β (6HIH)	McP460 (6HIU)	NeP460 (2J3E)	NeALP460 (6AMG)
Fe–His N (Å)	2.10/2.09	2.06	2.25	2.15
Fe–water (Å)	—	2.43	—	—
Lys–heme (Å)	—	1.32/1.36	1.56	1.37

### Crystal structure of McCP-β: a P460-like fold with a hydrophobic distal pocket

The crystal structure of McCP-β was solved by SAD using the anomalous signal of the intrinsic heme Fe, and the structure determined to 1.61 Å resolution. McCP-β displays a highly β-sheet fold that is quite unlike any known cytochrome *c*′ structures (all previous examples being 4 α-helix bundles), yet very similar to McP460 (Fig. 1 and S3†). McCP-β exhibits a novel distal pocket structure relative to NeP460, McP460 and all cyt cp-α structures. The pocket is comprised of hydrophobic residues, with two aromatic Phe residues (Phe 32 and Phe 61) centrally located above the Fe in a ‘capping’ position well-suited to sterically influencing the binding of diatomic ligands (we name this structural feature the ‘Phe Cap’). This residue arrangement is different from that of the single Phe residue found to lie near-parallel to and above the heme in certain cyt cp-α structures. In contrast to the structures of cyt P460, no heme-protein crosslink is observed in the McCP-β structure and the equivalent residue, Phe 61, now forms part of the Phe cap. The proximal pocket contains the conserved haem ligand His 123 and an aromatic Phe 133 residue (oriented perpendicular to the imidazole plane), analogous to all reported cyt P460 structures this arrangement differs from that of cyts cp-α in which a conserved positive, polar residue (Arg or Lys) lies adjacent to the His ligand. The McCP-β structure also shows that the His ligand forms a H-bond to the carbonyl of Tyr 99, analogous to the situation in McP460 (Fig. 1). We note that the His ligands of NeP460 and NsALP460 are also H-bonded to a peptide carbonyl (albeit from a Met residue).

The thermal stability difference between McCP-β and McP460 may be considered in relation to their respective structures. McP460 has a significantly more hydrophilic pocket, which may allow water ingress and aid in denaturation. McCP-β in contrast is highly hydrophobic, with water molecules excluded from the heme region.

### Spectroscopic characterization and thermostability

**Fig. 2:**
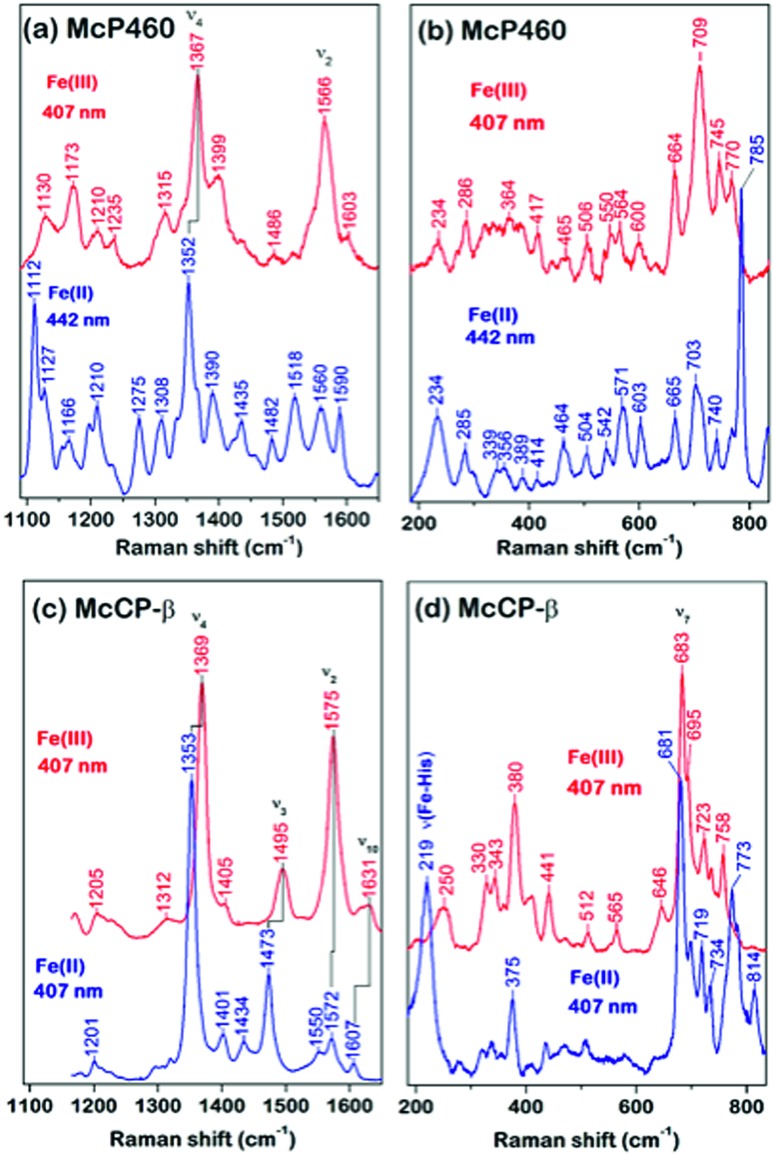
RR spectra of ferric and ferrous McP460 in the high-frequency (a) and low frequency (b) regions; RR spectra of ferric and ferrous McCP-β in the high-frequency (c) and low-frequency (d) regions. Data obtained at room temperature using either 407 nm or 442 nm excitation as indicated. Solutions contained ∼150 μM protein (in haem) in pH 7.0 buffer (50 mM MOPS).

As-isolated Fe(iii) McP460 (pH 7.0) exhibits a Soret absorption band at 419 nm, with a shift to the characteristic 460 nm band in the Fe(ii) state (Fig. S4, Table S1†). The *λ*_max_ value for the Fe(iii) state is significantly blue-shifted relative to that of NeP460 (434–440 nm) and NsALP460 (440 nm) (Table S1†), a fact that we attribute to the presence of a distal H_2_O ligand in McP460. Room-temperature Resonance Raman (RR) spectra of fresh McP460 were obtained at room temperature using laser excitations at 407 nm (for the ferric state) and 442 nm (for the ferrous state) (Fig. 2a and b). A notable feature of the P460 RR spectra (particularly apparent in the Fe(ii) state) is the increased number of observable porphyrin vibrations relative to McCP-β (Fig. 2c and d), a consequence of the Lys cross-link which lowers the porphyrin symmetry. Although the porphyrin modes of P460 hemes have yet to be systematically assigned, the most intense porphyrin RR bands of the Fe(iii) (1367 cm^–1^) and Fe(ii) (1352 cm^–1^) states are characteristic of *ν*_4_ redox marker modes, with the 1566 cm^–1^ band of the Fe(iii) state resembling the *ν*_2_ spin-state marker mode (Fig. 2a, Table S2†). The low frequency RR region also contains numerous RR bands (Fig. 2b). Unlike other heme proteins in which the most intense band in the 200–700 cm^–1^ region is the *ν*_7_ mode (typically observed near 680 cm^–1^), Fe(iii) McP460 exhibits its most intense low-frequency RR band at 709 cm^–1^, while that of the Fe(ii) state is at 785 cm^–1^. Recent RR measurements of Fe(iii) NeP460 (using 458 nm excitation) also showed a complex RR spectrum.^15^ However, in contrast to McP460, the RR modes of NeP460 exhibited different intensity patterns, and no porphyrin marker bands could be assigned. Neither could porphyrin marker bands be identified in RR spectra of the Fe(ii) P460 fragment of NeHAO.^26^ While the exact cause of these differences is unclear, it likely results from differences in porphyrin conformation/symmetry between the different P460 cofactors (*vide infra*).

A low temperature EPR analysis of McP460 showed a spectrum representative of a single high spin species (Fig. S5†) with *g*_1_ = 6.18, *g*_2_ = 5.57 and *g*_3_ = 1.99 resembling that of as isolated enzyme,^27^ Table S3.† In our hands McP460 undergoes a spectroscopic shift if not snap-frozen relatively soon following the final stage of purification, with the ‘degraded’ variant exhibiting absorption maxima at 415 nm in the Fe(iii) form and 455 nm in the Fe(ii) state, with a colour change from green (fresh protein) to brown (degraded protein) (Fig. S6†). This process also affects the RR spectra (Fig. S7†) and is discussed in more detail in the ESI.† We note here that all data presented are, unless stated otherwise, for the fresh, green form of McP460.

UV-visible absorption bands of as-isolated recombinant McCP-β (Fig. S8, Table S1†) are similar to those previously reported for the native protein,^6^ with a Soret maximum at 399 nm (and weak shoulder at ∼379 nm), together with an unresolved α/β band near 500 nm and a CT1 band at ∼640 nm, the latter characteristic of 5c high-spin (5cHS) ferric heme. The spectroscopic properties of Fe(iii) McCP-β remain essentially unchanged over the pH range 4.0–10.0 (Fig. S9†). Resonance Raman (RR) spectra of Fe(iii) McCP-β (obtained with 407 nm excitation) reveal porphyrin marker band frequencies: *ν*_4_ (1369 cm^–1^), *ν*_3_ (1495 cm^–1^), *ν*_2_ (1575 cm^–1^), *ν*_10_ (1631 cm^–1^) (Fig. 2c upper trace, Table S2†). The *ν*_3_ and *ν*_10_ spin-state frequencies are close to those attributed to the 5cHS Fe(iii) states of cyts cp-α (*ν*_3_ ∼1490–1494 cm^–1^ and *ν*_10_ ∼1625–1629 cm^–1^).^28^ The EPR spectrum of Fe(iii) McCP-β at pH 9 shows a mixture of two high spin species (Fig. S10, Table S3†) consistent with previous reports.^29^

Reduction of Fe(iii) McCP-β with dithionite yields the Fe(ii) state which exhibits a Soret maximum at 431 nm and a broad α/β band centered at 552 nm (Fig. S8, Table S1†). RR spectra of Fe(ii) McCP-β (obtained with 407 or 442 nm excitation) resemble those of Fe(ii) cyts cp-α, and have frequencies characteristic of 5cHS Fe(ii) hemes: *ν*_4_ (1353 cm^–1^), *ν*_3_ (1473 cm^–1^), *ν*_2_ (1572 cm^–1^), *ν*_10_ (1607 cm^–1^) (Fig. 2c lower trace, Table S2†). In the low frequency region, the *ν*(Fe–His) vibration of Fe(ii) McCP-β is evident at 219 cm^–1^, which is ∼10–15 cm^–1^ lower than for Fe(ii) cyt cp-α proteins,^30^ suggesting that McCP-β has a weaker proximal bond. The Fe–His bond strength in McCP-β may have important ramifications for its proposed heme–NO binding role, and in particular its ability to form 5c *vs.* 6c {FeNO}^7^ complexes. It should also be noted that McCP-β does not degrade in the same fashion as McP460.

**Fig. 3:**
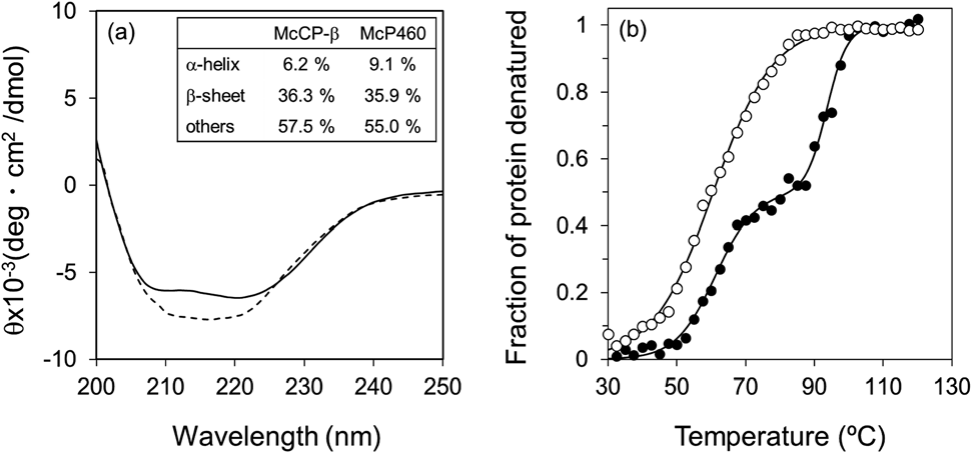
CD spectra (a) of McCP-β (dashed line) and McP460 (solid line). (b) Thermal denaturation curves for McCP-β (closed circles) and McP460 (open circles) as measured by CD. Representative normalized raw data plots are shown at the temperature interval of 2.5 °C.

The circular dichroism (CD) spectra of McP460 and McCP-β showed negative peaks from 205 to 225 nm. From the spectral data, the ratios of α-helix : β-sheet : others in McP460 and McCP-β were estimated as 9 : 36 : 65 and 6 : 36 : 58 (Fig. 3a), respectively. Previously reported ratios for NeP460 also estimated a high level of β-sheet, although the amount of α-helix was estimated to be much higher than that of McP460 and McCP-β.^5^ This indicates that these proteins have similar content of β-sheet as observed in these crystal structures.

The thermal stability of the two proteins was characterised by CD ellipticity changes at 222 nm (Fig. S11†). The melting temperature (*T*_m_) values obtained were 58.4 ± 2.8 °C for McP460 and 64.4 ± 3.2 and 94.1 ± 0.9 °C for McCP-β (Fig. 3b), indicating that the latter has a two-step transition during its thermal denaturation and more stable than the former. The unfolding transitions are much broader for McP460 and McCP-β as is typical for β-sheet proteins in comparison to those of cyts cp-α.^31^,^32^ We note for context that the optimum growth temperature of *M. capsulatus* (Bath) is 45 °C.^33^

### Evolution of cyt P460 and cyt cp-β proteins

**Fig. 4:**
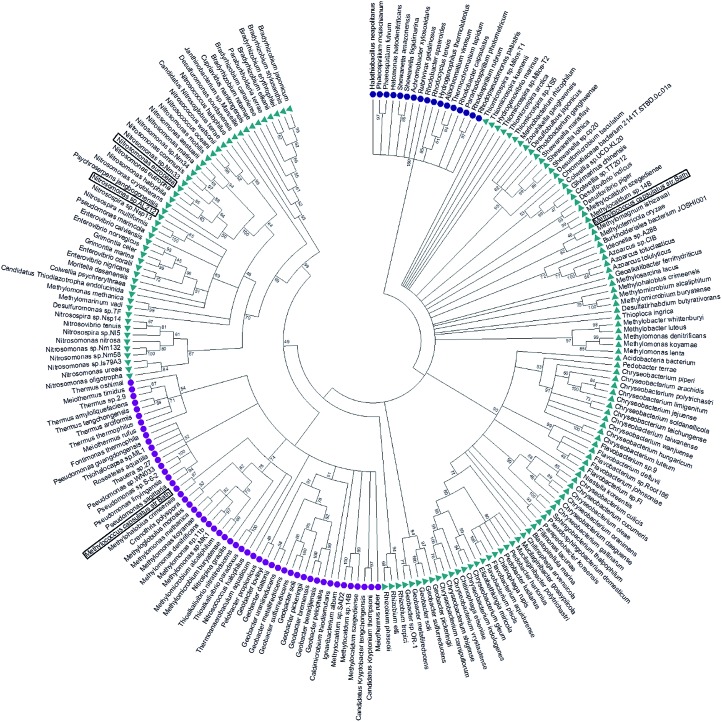
Maximum likelihood tree of cyt cp-α (blue circles), cyt cp-β (purple circles) and cyt P460 (green triangles) sequences. The cyt cp-α genes are on a separate branch to the cyt cp-β and the cyts P460 genes. The cyt P460 family displays a paraphyletic structure, with a monophyletic cyt cp-β nested within suggesting that the cyt cp-β evolved from the cyt P460. Image was prepared using Mega7.^54^

McCP-β has 31% sequence identity to McP460 and also possesses 18% identity to cyt P460 from *N. europaea*. In contrast, McCP-β has very low sequence identity to the well-characterised cyt cp-α proteins. A maximum likelihood phylogenetic tree displays maximum support for the branch separating a clade containing the cyt cp-α genes from a group comprising the cyt cp-β and the cyts P460 genes (Fig. 4). The cyt P460 family shows a paraphyletic structure, with a monophyletic cyt cp-β (44% bootstrap support) nested within. The group of P460 sequences which includes McP460 is not closely related to the cyts cp-β clade. Another group of P460 sequences, which contains both NeP460 and NsALP460, are more closely related to the cyts cp-β (49%) instead. It can be inferred from this result that the cyt cp-β proteins evolved from the cyt P460, as the latter are nested several nodes within the cyt P460, although these nodes have moderate bootstrap supports. Notably this topology is different to the one recovered in a previous study;^5^ this may be explained by the use of a much larger dataset in our study, together with more sophisticated phylogenetic methods (*e.g.*, maximum likelihood instead of neighbour joining). There are 14 organisms within the aligned sequences which possess both a cyt P460 and a cyt cp-β. These genes are not close to each other on the genome, arguing against a recent gene tandem duplication event. In all but two of these (*Nitrosococcus halophilus* and *Methylomonas methanica*) the cyt P460 sequences are placed on a clade that is not related to the cyts cp-β genes.

Alignment of all 144 cyt P460 sequences showed full conservation of only 5 residues (Trp 24, Gly 73, Lys 78, Lys 115 and Trp 17 in McP460) along with the CXXCH motif. The conserved lysine cross link in cyts P460 (Lys 78 in McP460) has been shown to be important in relation to the catalytic ability of the protein,^25^ whilst most of the other conserved residues are, as in the P460 of *N. europaea*, clustered at the base of the monomer.^12^ The group of cyts P460 that appear more closely related to the cyts cp-β have a higher level of homology to each other with an additional 20 conserved residues. The residues present in the distal pocket of NsALP460 (Phe 76 and His 80), are highly conserved within this subgroup (being present in 45 and 39 (out of 50) of the sequences respectively) with the alternative residues having similar properties.

The other group of cyts P460 (containing McP460) show five further conserved residues (Arg 50, Asn 55, Ala 58, Pro 71, Met 104 in McP460) when aligned together in comparison to the alignment of all 144 cyt P460 sequences. Two of these residues are positioned near the heme of McP460, Met 104 being to the side of the heme in the proximal pocket and Arg 50 sitting directly above the heme in the distal pocket. The type of residue in the distal pocket for this subgroup of P460 s appears reasonably conserved with the majority of them being charged, hydrophilic, polar residues. Arg 50 for example is present in 85 (out of 95) of these sequences. As previously shown,^5^ most of the cyt P460 and cyt cp-β sequences are from a wide range of proteobacteria.

### Structure–function relationships in cyt P460: implications for synthetic enzyme design

McP460 and NeP460 are well separated in the phylogenetic tree of P460 enzymes yet both are able to oxidise hydroxylamine, as can the unrelated enzyme HAO. In the mechanism proposed for hydroxylamine oxidation in NeP460,^14^ an initial Fe(iii)–NH_2_OH substrate complex undergoes a three electron oxidation to form a {FeNO}^6^ (Enemark–Feltham notation) intermediate *via* a 6c {FeNO}^7^ precursor. Nucleophilic attack of the {FeNO}^6^ species by a second NH_2_OH molecule, together with an additional one-electron oxidation, results in the formation of N_2_O product and the Fe(iii) heme resting state.

**Fig. 5:**
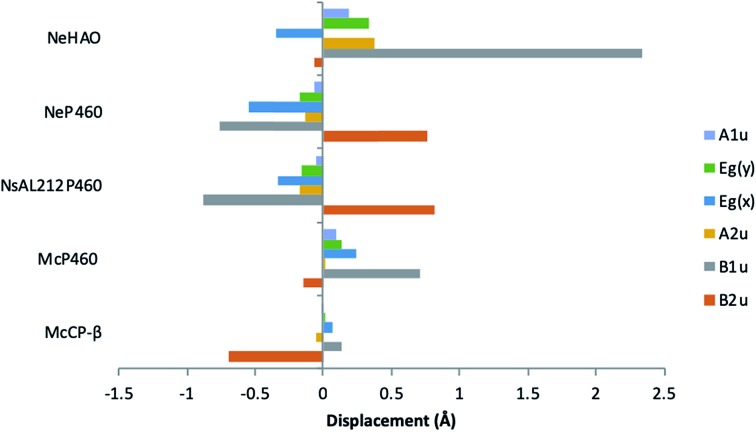
Out-of-plane displacements (minimal basis) for the hemes of McCP-βl (6HIH), McP460 (; 6HIU), NsALP460 (; 6AMG), *N. europaea* P460 (J2E3) and the P460 heme of *N. europaea* HAO (4FAS). Out of plane distortions are characterized in terms of displacements along the normal coordinates of the *D*_4h_-symmetric porphyrin macrocycle. NsALP460 and NeP460 display much higher overall distortions to both *M. capsulatus* (Bath) cytochromes. McP460 displays a high amount of ruffling (B1u) which has been proposed to be important in the function of the P460 cytochromes.^13^ In comparison to the other two cytochromes P460, McP460 does not have an equal amount of ruffling and saddling, a pattern that is more similar to that of the P460 heme of *N. europaea* HAO. McCP-β correspondingly does not display a high level of ruffling, although it does demonstrate a large amount of saddling (B2u). See also Table S5.†

The distinguishing structural feature of P460 cofactors is the Lys(Tyr) porphyrin cross-link. Notably, in NeP460, mutation of the cross-linking Lys to Arg, Ala or Tyr resulted in catalytically inactive proteins with spectroscopic properties similar to those of the cyts cp.^25^ It was recently proposed that the Lys-heme cross-link in NeP460 provides structural rigidity and prevents formation of an off-pathway 5c {FeNO}^7^ species. Out of plane heme deformations are another feature of P460 centers. Normal-coordinate structural decomposition (NSD) analyses of crystallographic data from NeP460 (J2E3) and NsALP460 (; 6AMG)^34^ revealed that these P460 heme cofactors have relatively high degrees of ruffling (B1u) and saddling (B2u) distortions,^13^ whereas the P460 center of NeHAO (4FAS) is distorted predominantly by ruffling. It has been proposed that a high degree of ruffling (rather than saddling) is a hallmark of P460 cofactors. Consistent with this proposal, NSD analysis of our McP460 structural data also reveal a high degree of ruffling with relatively little saddling, whereas McCP-β exhibits a degree of saddling comparable with NeP460 and NsALP460, but has very little ruffling (Fig. 5, Table S5†). It has been further proposed that heme properties such as lower reduction potentials^35^ and stronger bonding between the Fe and axial ligand^36^ are associated with increased heme ruffling, suggesting that these distortions are important to P460 function. In particular, a low reduction potential has been proposed as the reason why HAO and P460 avoid auto-reduction upon NO binding. In this context, we also note that McP460 has a significantly lower *E*° value (–380 to –300 mV) than that of McCP-β (–205 mV),^6^ although structural differences other than ruffling (*e.g.* axial heme environments) may also influence heme redox properties.

In addition to the presence of a porphyrin-Lys (or Tyr) cross-link, crystal structures of McP460, NeP460, and HAO show polar distal heme pockets with side-chains positioned to form hydrogen bonds with exogenous ligands. For example, in McP460, a 3.1 Å hydrogen bond is formed between Asp 102 and the heme-bound water molecule (Fig. 1). Other distal ligands relevant to the mechanism, such as hydroxylamine, NO or N_2_O would likely be in a position to form hydrogen bonds with several Arg or Asp residues within the pocket, Fig. 1. Interestingly, the recently determined structure of NsALP460 (PDB ; 6AMG) revealed a quite different distal pocket environment. The heme retains its P460 character with the presence of a Lys cross-link, but the distal pocket is far more hydrophobic with residue Phe 76 in a similar position to that of Phe32 in McCP-β. It is conceivable that NsALP460 could provide clues as to the evolutionary divergence between McCP-β and cyts P460, representing a half-way house situation. Testing of this hypothesis awaits the demonstration of enzymatic activity in NsALP460. We also note that a conserved Tyr residue in the heme pocket of HAO from AOBs has been proposed to play a key role in the catalytic mechanism.^37^ In methanotroph HAOs this residue is absent, leading to suggestions that the final stage of the HAO mechanism is arrested. In P460 structures, the equivalently located residue is an Arg (50 McP460 and 44 NeP460) or His (NsAL212-P460). The extent to which enzymatic activity in P460 heme proteins is related to distal pocket structure *versus* heme geometry will be addressed in future studies.

### Functional evolution of cyts cp-β: a possible NO binding role?

The availability of high resolution crystal structures of a cyt cp-β and a cyt P460 from the same organism allows the architecture of the two related proteins to be considered in a structure–function context independent of species differences. In contrast to McP460, there is no porphyrin-Lys cross-link or potential H-bonding residue to interact with heme-bound water or diatomic ligands in McCP-β, and indeed the heme is 5-coordinate in the resting state with a vacant distal heme binding site.

There is low sequence conservation within the cyt cp-β family, with 7 other residues in addition to the CXXCH motif being fully conserved across the 52 aligned cyt cp-β sequences (Trp14, Gly34, His36, Tyr39, Met84, Thr94, Trp97 in McCP-β). This is similar to the low conservation of sequence in cyts cp-α.^22^ Phe 32 is highly conserved within cyts cp-β, being present in all but one of the aligned sequences (*Meiothermus ruber*). Phe 61 is slightly less conserved, being present in 39 of the sequences, although all are hydrophobic residues. Within the proximal pocket, Tyr 131 is conserved in just over two thirds of the cyt cp-β sequences (36 out of 52), although all other residues are hydrophobic (either Phe or Leu). In addition, Phe 133 is conserved in 38 of the 52 cyt cp-β sequences.

No organisms identified in our study have both a cyt cp-β and a cyt cp-α (or indeed a cyt P460 and cyt cp-α). Between the two cyt cp families (alpha and beta) the only conservation is in the CXXCH motif. Nevertheless, the conserved ‘Phe cap’ in McCP-β is reminiscent of the occluding distal aromatic or aliphatic residue in cyt cp-α. In the case of cyts cp-α, the highly hydrophobic and sterically crowded distal pockets are important in their ability to discriminate between gaseous ligands by allowing small neutral ligands, such as NO and CO to bind to the heme.^16^ The cyt cp-α family of proteins occur in denitrifying, photosynthetic, methanotrophic, sulphur-oxidising and nitrogen-fixing bacteria. In each of these groups, the ability to bind NO may have functional importance. In denitrifiers, NO is produced by nitrite reductases (NiRs) and further reduced to N_2_O by nitric oxide reductase (NOR) enzymes. It has been proposed that cyt cp-α could act as a shuttle carrying this potentially dangerous free radical from NiR to the membrane-bound NOR. There is also evidence that cyt cp-α could itself act as an NO-reductase. Recent studies suggesting that HAO produces NO as an end product suggests a role for an NO-sequestering protein such as McCP-β to work in concert with HAO deal with excessive NO levels. The introduction of a hydrophobic distal heme environment by evolution in the cyt cp-β family is consistent with a functional requirement for the heme to selectively bind NO. We note that McCP-β has previously been shown to form complexes with both NO and CO.^6^ Notably, in contrast to cyts cp-α, in both McCP-β and McP460 the proximal His forms a H-bond to a main chain carbonyl which could influence any dissociation from Fe upon distal NO binding, a process shown to occur in cyts cp-α.

## Conclusion

We have characterised the closely related cytochrome P460 and cytochrome *c*′-β proteins from *Methylococcus capsulatus* (Bath). High resolution crystal structures reveal a highly similar tertiary structure between the two proteins but major structural differences around the heme pocket consistent with a switch of function between hydroxylamine oxidation (P460) and NO binding/sequestration (cyt cp-β). Interestingly, our structures reveal that the protein fold of McP460 is rather more similar to McCP-β than it is to NeP460, with the exception of the heme binding pocket where a number of localised structural alterations are associated with functional conversion. In particular, the lysine that forms the heme cross-link of McP460 has been replaced in McCP-β by a Phe residue that instead provides steric hindrance to distal ligand binding as part of the ‘Phe Cap’ motif. The heme pocket of McCP-β is much less solvent-exposed than in McP460 with a highly hydrophobic distal pocket with no water molecules observed within the McCP-β heme pocket. Phylogenetic analysis of the occurrence of these two proteins suggests an evolutionary path such that the cyt cp-β (*CytL* gene) proteins evolved from the cyts P460 (*CytS*) gene, likely in response to the need to capture excess nitric oxide.

## Materials and methods

### Expression and purification

The genes encoding McCP-β and McP460 (Accession numbers AAD39218 and AAU93287 from the NCBI RefSeq database) were commercially synthesised (Epoch Life Sciences) in a pBSK plasmid with restriction enzyme sites (EcoRI and XbaI respectively) flanking either end of the gene. The gene was cut from this plasmid and ligated into the expression vector pMMB503EH.^38^*E. coli* XL-1 Blue cells (Stratagene USA) were used for general cloning, and were routinely grown at 37 °C in Luria Broth (LB) medium or plates. When required, antibiotics were used at the following final concentrations: streptomycin (50 μg ml^–1^), chloramphenicol (20 μg ml^–1^). *E. coli* BL21(DE3) (Novagen) competent cells containing pEC86,^39^ a plasmid carrying chloramphenicol resistance and the ccmA-H genes from *E. coli* necessary for the maturation of c-type cytochromes, were transformed with the McCP-β containing pMMB503EH vector. Cells were grown in LB or Terrific Broth (TB) and shaken at 200 rpm at 37 °C. For protein production, IPTG was added in mid-log phase to a final concentration of 119 μg ml^–1^. Ferriprotoporphyrin IX chloride was added to a final concentration of 30 μg ml^–1^ after 5 h and 1 ml of a metal ion mix (2 mM Ni^2+^, 2 mM Co^2+^, 10 mM Zn^2+^, 10 mM Mn^2+^ and 50 mM Fe^3+^) was added after 24 h in a procedure adapted from ref. 40.

Cultures were grown for 96 h and cells harvested by centrifugation for 20 min at 4 °C and at 4000 rpm. Harvested cells were resuspended in 20 mM Tris–HCl pH 8, and disrupted by 2 passages through an EmulsiFlex (12 000 psi). The crude McCP-β extract was prepared by centrifugation at 18 000 rpm for 30 min followed by 24 h dialysis against ddH_2_O. The extract was then centrifuged at 18 000 rpm for 20 min and loaded onto a DEAE Sepharose Fast Flow anion-exchange column previously equilibrated with two column volumes of 20 mM Tris–HCl, pH 8. McCP-β was eluted using 10 mM NaCl and was further purified to homogeneity by gel filtration using a Sephadex G75 column. A final yield of 6.4 mg L^–1^ McCP-β was obtained. The crude McP460 extract was prepared by centrifugation at 18 000 rpm for 30 min followed by the addition of 70% ammonium sulfate. The extract was then centrifuged for 20 min at 18 000 rpm and loaded onto a phenyl-sepharose hydrophobic interaction column previously equilibrated with two column volumes of 0.5 M ammonium sulfate, 50 mM sodium phosphate pH 8. McP460 was eluted over a decreasing salt gradient and further purified to homogeneity by gel filtration using a Sephadex G75 column. The purity of the proteins was determined by SDS–PAGE analysis and by UV-vis spectroscopy using published molar absorptivity of McP460 and McCP-β heme Soret bands: Fe(iii) McP460: *ε*_419_ = 78.5 mM cm^–1^;^29^ Fe(iii) McCP-β: *ε*_400_ ∼ 70 mM cm^–1^.^6^

### Protein crystallisation and X-ray crystallography

Crystals of McCP-β were grown using the hanging drop vapour diffusion method at 18 °C. Brown cubic crystals were obtained after 3–5 days using 2 μl of 15 mg ml^–1^ protein in 0.1 M HEPES buffer, pH 7.5, with an equivalent volume of reservoir solution containing 0.01 M ZnSO_4_, 35% PEG 550 (v/v) and 0.1 M MES, pH 6.5. Prior to X-ray data collection crystals were cryoprotected by transfer to reservoir solution comprising mother liquor supplemented with 10% (v/v) glycerol and flash-cooled in liquid nitrogen. X-ray diffraction data were collected at beamline I03, Diamond Light Source, using a Pilatus 3 6 M detector. A dataset measured at a wavelength of 1.74 Å was used to solve the structure using the anomalous signal of the intrinsic heme Fe. All data were processed in Xia2 using XDS^41^ and AIMLESS.^42^ The initial model was built using Buccaneer.^43^ A high-resolution dataset was measured from a second MCCP crystal at a wavelength of 0.97 Å and refined from the starting model from the SAD experiment. Structures were refined by maximum likelihood methods using REFMAC5.^44^ Model building between cycles of refinement, including addition of water molecules and ligands was performed in Coot,^45^ and the quality of the structures was monitored using the MOLPROBITY^46^ and the JCSG Quality Control servers. Data collection and refinement statistics are shown in Table 1. Crystals of McP460 were grown using the hanging drop vapour diffusion method at 18 °C. Green orthorhombic crystals were obtained after 4–5 days using 2 μl of 15 mg ml^–1^ protein in 20 mM Tris buffer, pH 8, with an equivalent volume of reservoir solution containing 0.1 M Tris, 2.4 M ammonium sulfate pH 8. Prior to X-ray data collection crystals were cryoprotected by transfer to reservoir solution comprising mother liquor supplemented with 1.7 M sodium malonate and flash-cooled in liquid nitrogen. Data were measured at beamline I04-1, Diamond Light Source at a wavelength of 0.979 Å, processed using DIALS and the structure solved using Auto-Rickshaw,^47^ with an initial SAD electron density map being used as the target for molecular replacement with the structure of NeP460 [PDB ; 2je3]^12^ as the search model. Model building was completed in Arp–Warp^48^ and subsequently refined and validated as described for McCP-β above. Protein Data Bank accession numbers are given in Table 1.

### Spectroscopic analysis

A Varian Cary 50 spectrophotometer was used to measure UV-visible absorption spectra. Reduction of McCP-β and McP460 to the ferrous state was achieved through addition of excess sodium dithionite under anaerobic conditions.

The EPR spectra were measured on a Bruker EMX EPR spectrometer (X band) at 10 K. A spherical high-quality Bruker resonator ER 4122 SP 9703 and an Oxford Instruments liquid helium system were used to measure the low temperature EPR spectra. Wilmad SQ EPR tubes (Wilmad Glass) were filled with the McCP-β or McP460 solutions and frozen in methanol kept on dry ice. The tubes were then transferred to liquid nitrogen. The spectra were measured at the following instrumental conditions: microwave frequency *ν*_MW_ = 9.4668 GHz; microwave power *P*_MW_ = 3.17 mW; modulation frequency *ν*_M_ = 100 kHz; modulation amplitude *A*_M_ = 5 G; scan rate *v* = 22.6 G s^–1^; time constant *τ* = 81.92 ms; conversion time, at a 2048 data point scan range, *t*_conv._ = 81.92 ms.

Protein solutions for RR measurements (100–150 μM in heme) were prepared in 50 mM MOPS buffer (pH 7.0) containing 0.10 M NaCl. Samples of as-isolated Fe(iii) proteins were transferred to glass capillary tubes for RR measurements. Reduced proteins were prepared with O_2_-free solutions inside an anaerobic glove box (using excess dithionite to reduce the Fe(iii) proteins to the Fe(ii) states) and transferred to septum-sealed glass capillary tubes for RR measurements. RR spectra (room-temperature, 90°-scattering geometry) were recorded on a custom McPherson 2061/207 spectrograph (100 μm slit width, 0.67 m focal length, 2400 grooves/mm holographic grating) equipped with a Princeton Instruments liquid N_2_-cooled (LN-1100PB) CCD detector. Excitation wavelengths were provided by Kr ion laser (406.7 nm) and a He–Cd laser (441.6 nm). RR spectra were recorded for periods of 1–3 min using laser powers of 10–30 mW (measured at sample). An indene standard was used to calibrate Raman shifts to an accuracy of ±1 cm^–1^. The identity of RR samples was confirmed by UV-vis spectroscopy before and after exposure to the laser beam using a modified Cary 50 spectrophotometer.

A JASCO J-820 CD spectrometer was used for circular dichroism (CD) spectrum measurement. The spectra (200–250 nm) of the McCP-β and McP460 proteins (20 μM) in 25 mM sodium acetate buffer pH 5.0 at 25 °C were measured. The components of the protein secondary structures were estimated from the CD spectra using BeStSel program.^49^,^50^

Thermal stability of McCP-β and McP460 was also measured by CD spectra in a pressure-proof cell compartment (Jasco, Tokyo) attached to the CD spectrometer. The protein solutions (20 μM) were dialysed against 25 mM sodium acetate buffer pH 5.0 at 25 °C. The temperature-dependent CD ellipticity changes at 222 nm were monitored in a cuvette of 1 mm path length. The CD values were recorded from 25 to 120 °C at intervals of 0.5 °C at a heating rate of 1.0 °C min^–1^ under the 0.9 MPa pressure conditions. The raw data were subjected to nonlinear least-squares fitting as described previously.^51^ The data points were corrected for the slopes of the baselines for the native and denatured forms and were normalized to calculate the fraction of protein denatured. The value for fraction denatured was plotted as a function of temperature, and the resulting thermal denaturation curves were used to determine the temperature at the midpoint of the transition (*T*_m_) during protein denaturation.

### Bioinformatics

Protein BLAST^52^ was performed May 2018 against NCBI GenBank using as query the sequences *Methylococcus capsulatus* (Bath) cytochrome *c*′ (WP_010961620.1), *Methylococcus capsulatus* (Bath) P460 (WP_010959872.1), *Nitrosomonas europaea* P460 (WP_011110663.1) and *Alcaligenes xylosoxidans* cytochrome *c*′ (WP_088139370.1). After removal of redundant sequences, full length protein sequences were aligned using ClustalW.^53^ Any sequences that did not have the cross-linking Lys residue were removed from the list of cyts P460. A total of 214 identified cytochrome *c*′ (69 sequences) and P460 (145 sequences) protein sequences were used in the alignments. Maximum likelihood trees were constructed using RAxML version 8.2.10 54 with the evolutionary model LG + Gamma + Invariants, and the support for each was assessed with 1000 rapid bootstrap replicates. The resulting tree was annotated using MEGA7.^55^

### Normal-coordinate structural decomposition

Out of plane distortions of the heme were analysed using an online normal coordinate structural decomposition script (http://mliptak.w3.uvm.edu/nsd.html) derived from the normal-coordinate structural decomposition procedure.^34^,^56^

## Conflicts of interest

There are no conflicts to declare.

## Supplementary Material

Supplementary information
