# Correction: One fold, two functions: cytochrome P460 and cytochrome c′-β from the methanotroph *Methylococcus capsulatus* (Bath)

**DOI:** 10.1039/c9sc90032b

**Published:** 2019-02-13

**Authors:** Hannah R. Adams, Callie Krewson, Jenny E. Vardanega, Sotaro Fujii, Tadeo Moreno-Chicano, Yoshihiro Sambongi, Dimitri Svistunenko, Jordi Paps, Colin R. Andrew, Michael A. Hough

**Affiliations:** a School of Biological Sciences , University of Essex , Wivenhoe Park , Colchester , Essex CO4 3SQ , UK . Email: mahough@essex.ac.uk; b Department of Chemistry and Biochemistry , Eastern Oregon University , La Grande , Oregon 97850 , USA . Email: candrew@eou.edu; c Graduate School of Biosphere Science , Hiroshima University , Kagamiyama 1-4-4, Higashi-Hiroshima , Hiroshima , 739-8528 , Japan

## Abstract

Correction for ‘One fold, two functions: cytochrome P460 and cytochrome c′-β from the methanotroph *Methylococcus capsulatus* (Bath)’ by Hannah R. Adams *et al.*, *Chem. Sci.*, 2019, DOI: ; 10.1039/c8sc05210g.



## 

The authors regret an error in the listing of one of the authors, Tadeo Moreno-Chicano, in the original manuscript. The corrected list of authors and affiliations for this paper is as shown above.

The Royal Society of Chemistry apologises for these errors and any consequent inconvenience to authors and readers.

