# Correction: One fold, two functions: cytochrome P460 and cytochrome c′-β from the methanotroph *Methylococcus capsulatus* (Bath)

**DOI:** 10.1039/c9sc90185j

**Published:** 2019-09-09

**Authors:** Hannah R. Adams, Callie Krewson, Jenny E. Vardanega, Sotaro Fujii, Tadeo Moreno-Chicano, Yoshihiro Sambongi, Dimitri Svistunenko, Jordi Paps, Colin R. Andrew, Michael A. Hough

**Affiliations:** a School of Biological Sciences , University of Essex , Wivenhoe Park , Colchester , Essex CO4 3SQ , UK . Email: mahough@essex.ac.uk; b Department of Chemistry and Biochemistry , Eastern Oregon University , La Grande , Oregon 97850 , USA . Email: candrew@eou.edu; c Graduate School of Biosphere Science , Hiroshima University , Kagamiyama 1-4-4, Higashi-Hiroshima , Hiroshima , 739-8528 , Japan

## Abstract

Correction for ‘One fold, two functions: cytochrome P460 and cytochrome c′-β from the methanotroph *Methylococcus capsulatus* (Bath)’ by Hannah R. Adams *et al.*, *Chem. Sci.*, 2019, **10**, 3031–3041.



## 

The authors regret that there was an error in the following sentence of the conclusions in this manuscript:

“Phylogenetic analysis of the occurrence of these two proteins suggests an evolutionary path such that the cyt cp-β (*CytL* gene) proteins evolved from the cyts P460 (*CytS*) gene, likely in response to the need to capture excess nitric oxide.”

The correct sentence should read as follows:

“Phylogenetic analysis of the occurrence of these two proteins suggests an evolutionary path such that the cyt cp-β (*CytS* gene) proteins evolved from the cyts P460 (*CytL* gene), likely in response to the need to capture excess nitric oxide.”

The Royal Society of Chemistry apologises for these errors and any consequent inconvenience to authors and readers.

