# Supplementary material for: One fold, two functions: cytochrome P460 and cytochrome c′-β from the methanotroph Methylococcus capsulatus (Bath)
Source: Chem Sci. 2019 Jan 21;10(10):3031–41. doi: 10.1039/c8sc05210g (PMC6427953; doi:10.1039/c8sc05210g)
Supplement: Supplementary file 1 [file SC-010-C8SC05210G-s001.pdf]

Electronic Supplementary Material (ESI) for Chemical Science.
This journal is © The Royal Society of Chemistry 2019

SUPPLEMENTARY ONLINE INFORMATION

Table S1: UV-vis absorption maxima for cyts cp-β and P460

			λ_{max} (nm)			ref
**Fe(III)**						
	McCP-β	379sh	399	499	640	tw
			401	502	638	1
	McP460		419		628	tw
			419			2
	NeP460		434	510sh	540	3
		414sh	440	570	627	4
	NsALP460		440	576	628	5
**Fe(II)**						
	McCP-β	372	431	552		tw
			433	552		1
	McP460		460	574	684	tw
	NeP460		462	660	668	3

Abbreviations: tw, this work.

Table S2: Spin states and Porphyrin marker RR frequencies (cm^{-1}) of Cyts P460 and Cyt cp-β.

protein	Redox State	spin state	ν_4	ν_3	ν_2	ν_{10}	ref
Cyt P460							
McP460 (wt)	Fe(III)	6cHS	1369	na	1569	na	tw
	Fe(II)	5cHS	1352	na	na	na	tw
NeP460 (wt)	Fe(III)	HS	na	na	na	na	6
NeP460 (K70Y)	Fe(III)	HS	1372	1501	na	na	6
	Fe(II)	5cHS	1356	1473	na	na	6
Cyt cp-β							
McCP-β	Fe(III)	5cHS	1369	1495	1575	1631	tw
	Fe(II)	5cHS	1353	1473	1572	1607	tw

Abbreviations: 5c, five-coordinate; 6c six-coordinate; HS high-spin, na, not assigned; tw, this work.

Table S3. EPR parameters for cyts P460 and c'-β.

Protein	pH	Temp (K)	$g_{\perp}$		$g_{\parallel}$	Ref
			g_1	g_2	g_3	
			P460			
McP460	7	10	6.18	5.57	1.99	this work
NeP460[a]	7	4	5.91	5.63	1.99	[7]
NeP460[b]			6.57	5.09	1.97	[4]
NsALP460			6.39	5.13	1.97	[5]
McP460	8.2	8	6.26-6.18	<5.43	2.00	[2]
			Cyt cp-β			
McCP-β (major)	9	10	6.25	5.33	1.99	this work
McCP-β (minor)	9	10	5.94	5.58	1.99	this work
McCP-β (major)	8	8	6.29	5.34	2.00	[2]
McCP-β (major)	4	8	5.97	5.36	2.00	[2]

a. Enzyme as isolated from the original organism; b. recombinant enzyme.

Table S4 Geometric Parameters Obtained from McP460 and the *Nitrosomonas sp. AL212* Cyt P460 Crystal Structure

	McP460 (A, B)	NsALP460 (A/B)
C2−C3	1.42 Å (1.42Å)	1.34 Å (1.33 Å)
C3−C4	1.42 Å (1.41Å)	1.30 Å (1.32 Å)
C7−C8	1.43 Å (1.42Å)	1.35 Å (1.32 Å)
C8−C9	1.44 Å (1.42Å)	1.35 Å (1.31 Å)
C12−C13	1.45 Å (1.43Å)	1.39 Å (1.39 Å)
C13−C14	1.48 Å (1.45Å)	1.41 Å (1.40 Å)
C17−C18	1.43 Å (1.40Å)	1.39 Å (1.33 Å)
C18−C19	1.43 Å (1.43Å)	1.30 Å (1.35 Å)
C13−NLys	1.35 Å (1.33Å)	1.37 Å (1.37 Å)
∠C2−C3−C4	128.3 (129.1)	133.4° (132.8°)
∠C7−C8−C9	126.1 (131.1)	130.1° (135.5°)
∠C12−C13−C14	123.7 (123.1)	123.0° (121.8°)
∠C17−C18−C19	123.0 (129.1)	127.5° (123.6°)
∠C12−C13−NLys	110.2 (97.7)	116.0° (118.1°)
∠C14−C13−NLys	123.8 (119.4)	120.9° (118.9°)

(to other Lys conf
A 129.3 / 105.6
B 118.2/117.7

Table S5. Normal-Coordinate Structural Decomposition for the hemes of McP460 (6HIU), McCP-β (6HIH), NsALP460 (6AMG), NeP460 (J2E3) and the P460 heme of NeHAO (4FAS), showing the level of deviation from planarity of the heme from saddling (B2u), ruffling (B1u), doming (A2u), waving (Eg(x) and Eg(y)) and propellering (A1u). Doop refers to the overall out-of-plane distortions (in Ångstroms).

Protein	Basis	Doop	B2u	B1u	A2u	Eg(x)	Eg(y)	A1u
McP460	Min.	0.774	-0.147	0.704	0.008	0.240	0.135	0.092
	Comp.	0.798	0.147	0.712	0.082	0.264	0.160	0.092
McCP-β	Min.	0.705	-0.691	0.131	-0.048	0.064	0.019	-0.016
	Comp.	0.718	0.702	0.131	0.056	0.064	0.037	0.021
NsALP460	Min.	1.271	0.819	-0.882	-0.172	-0.339	-0.154	-0.051
	Comp.	1.296	0.822	0.886	0.253	0.345	0.195	0.054
NeP460	Min.	1.224	0.761	-0.756	-0.130	-0.548	-0.171	-0.062
	Comp.	1.250	0.782	0.766	0.142	0.558	0.172	0.066
NeHAO	Min.	2.428	-0.070	2.346	0.371	-0.342	0.328	0.189
	Comp.	2.456	0.140	2.355	0.385	0.370	0.379	0.212

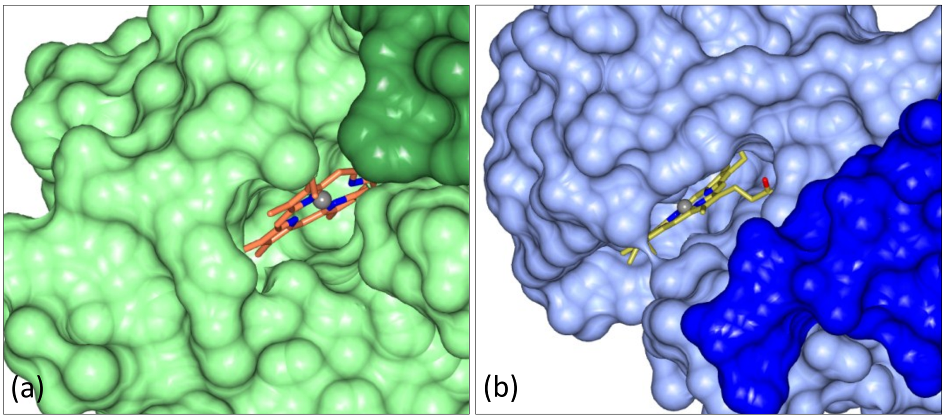

Figure S1. (A) Accessibility of the heme within McP460 monomer (B) Accessibility of the heme within McCP-β monomer, light and dark colours represent each monomer.

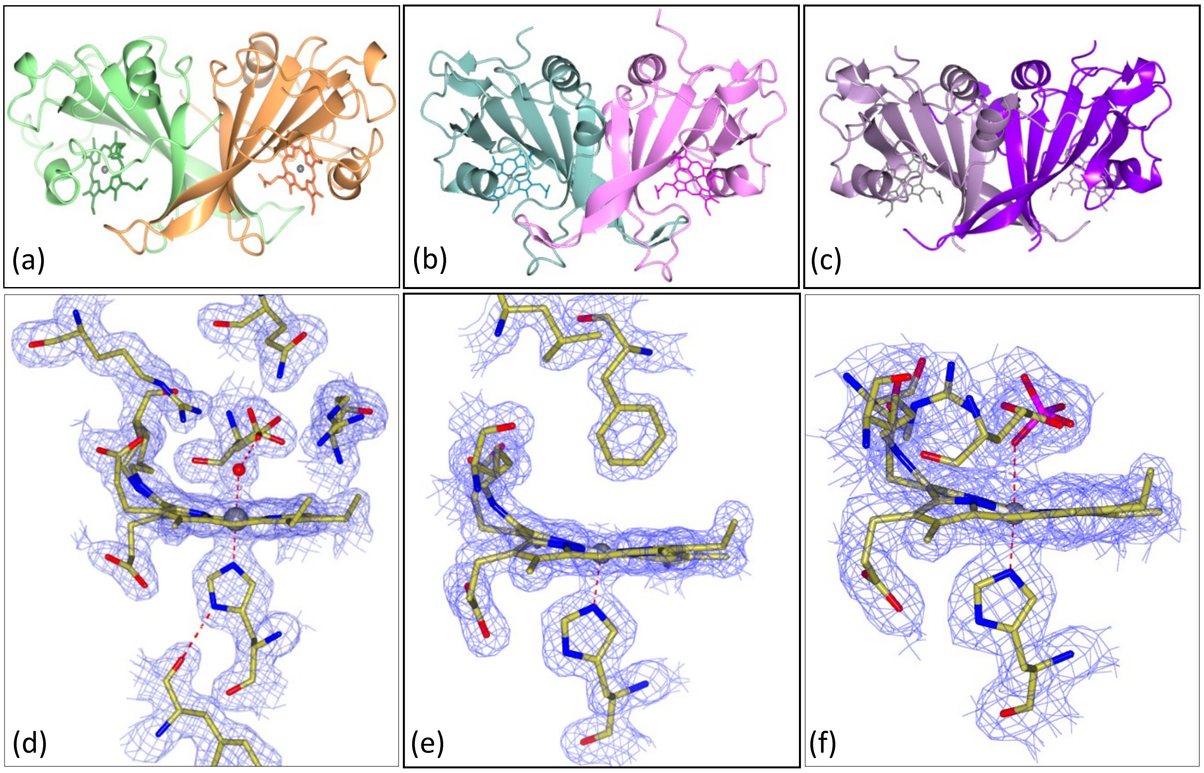

Figure S2. The homodimeric structures of (a) McP460, (b) NsALP460 and (c) NeP460 showing the predominantly β-sheet fold; (d) the heme environment of McP460 with the distal water ligand and hydrophilic, charged pocket with several Arg and Asp residues in a position to interact with bound substrates (e) the heme environment of NsALP460 displaying a much more hydrophobic distal pocket and (f) the heme environment of NeP460.

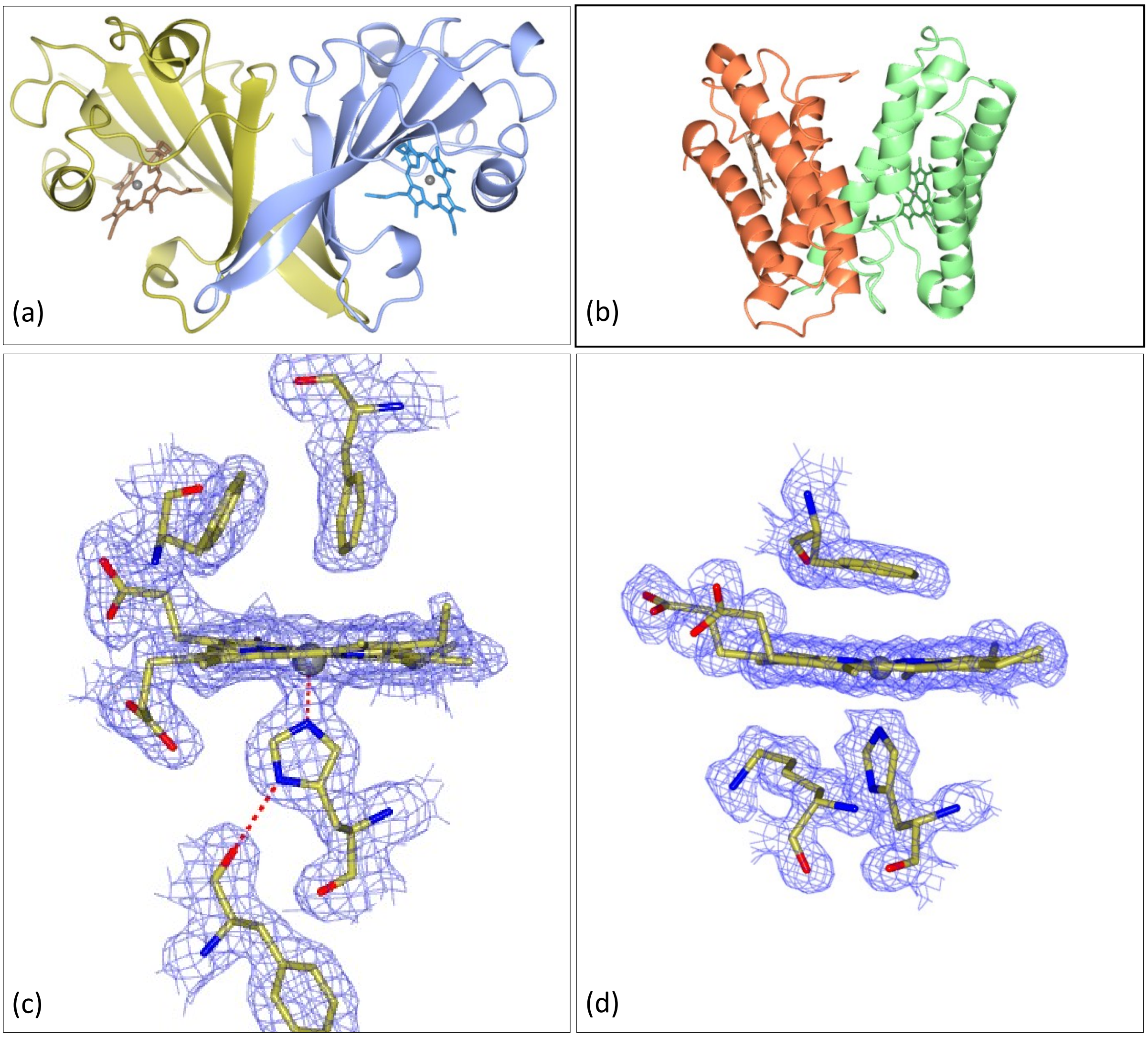

Figure S3. The homodimeric structures of (a) McCP-β showing the predominantly β-sheet fold and (b) SFCP (PDB 4ULV) displaying the typical alpha helical bundle; (c) the heme environment of McCP-β with the 'Phe Cap' of Phe 32 and Phe 61 sitting above the heme in the distal pocket (d) the heme environment of SFCP with Phe 16 lying near parallel to the heme in the distal pocket.

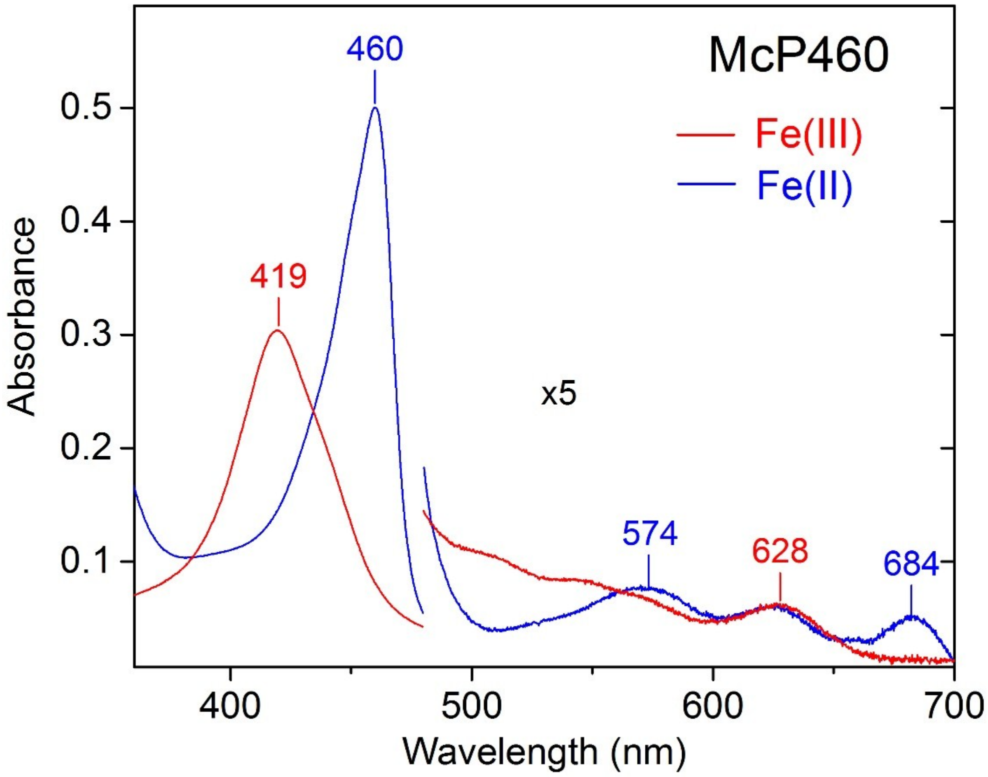

Figure S4: UV-vis Absorption Spectra of Fe(III) (red trace) and Fe(II) (blue trace) forms of McP460 pH 7.0. As-isolated Fe(III) McP460 exhibits a Soret absorption band at 419 nm, with a shift to the characteristic 460 nm band in the Fe(II) state

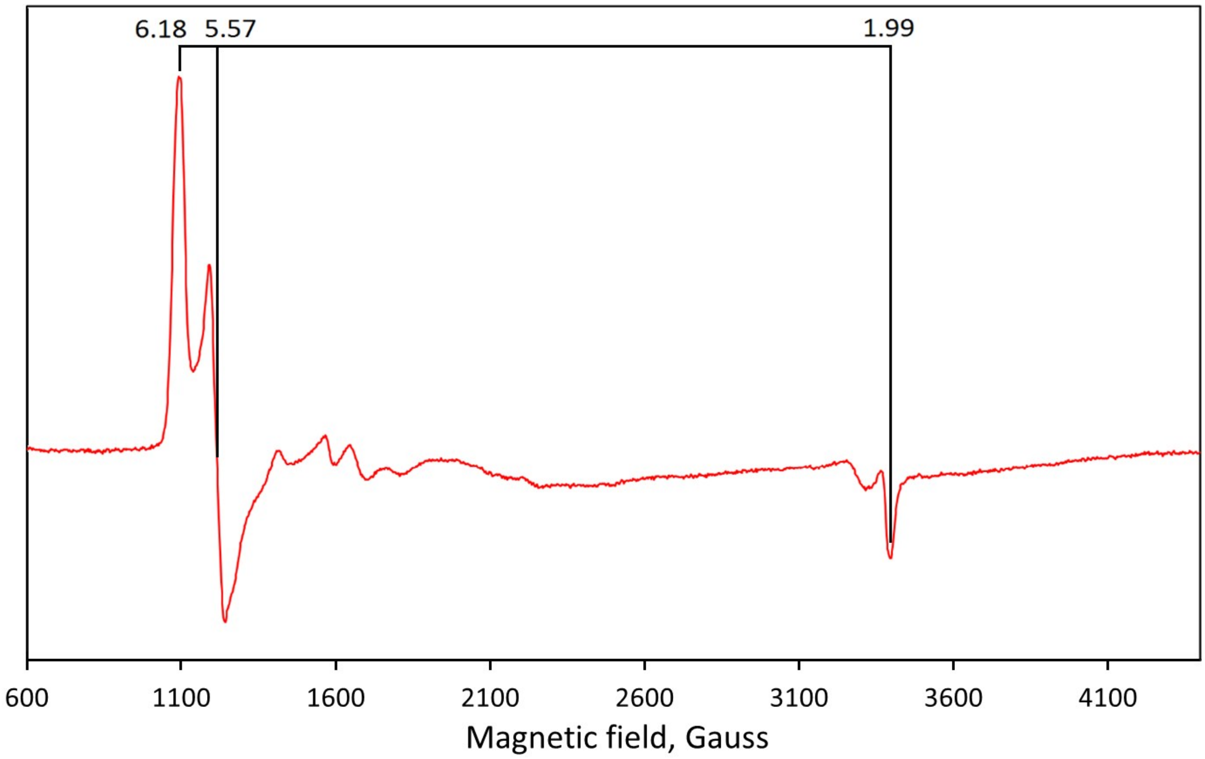

Figure S5: X-band EPR spectrum of McP460 at pH 7. The spectrum with g values of 6.18, 5.57 and 1.99 resembles that of NeP460[7].

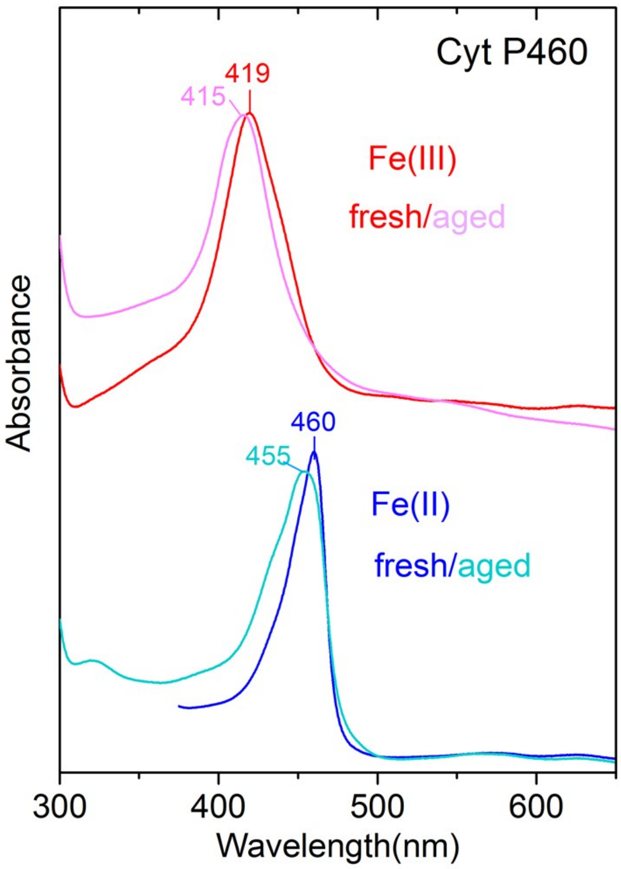

Figure S6. Effect of sample aging on the UV-vis absorption spectra of McP460.

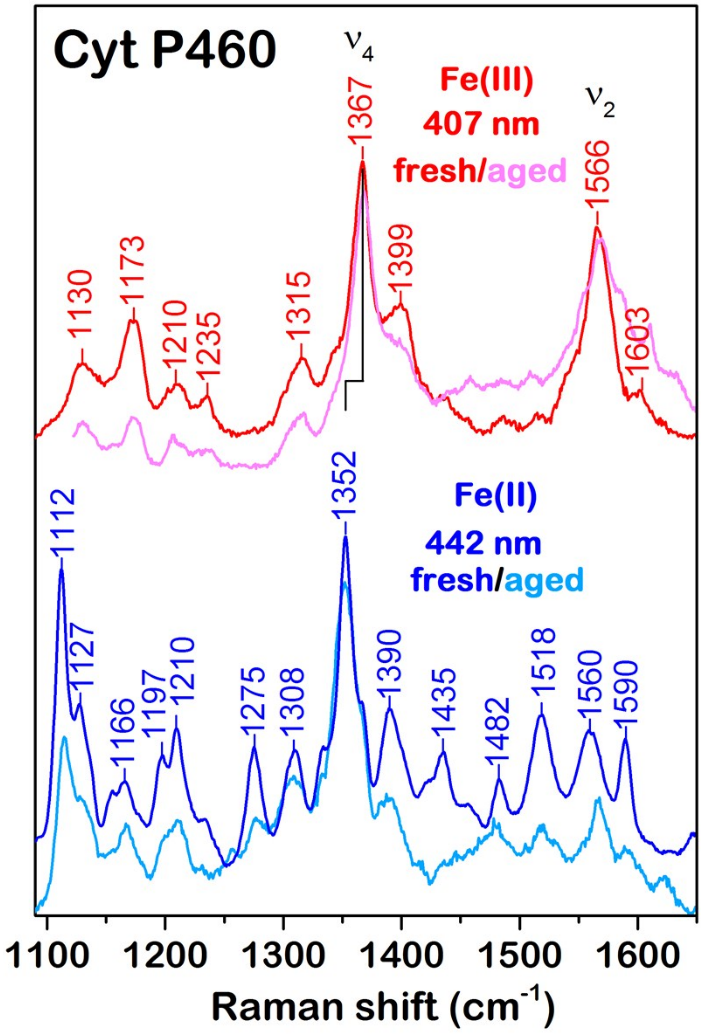

Figure S7. Effect of sample aging on the RR spectra of McP460.

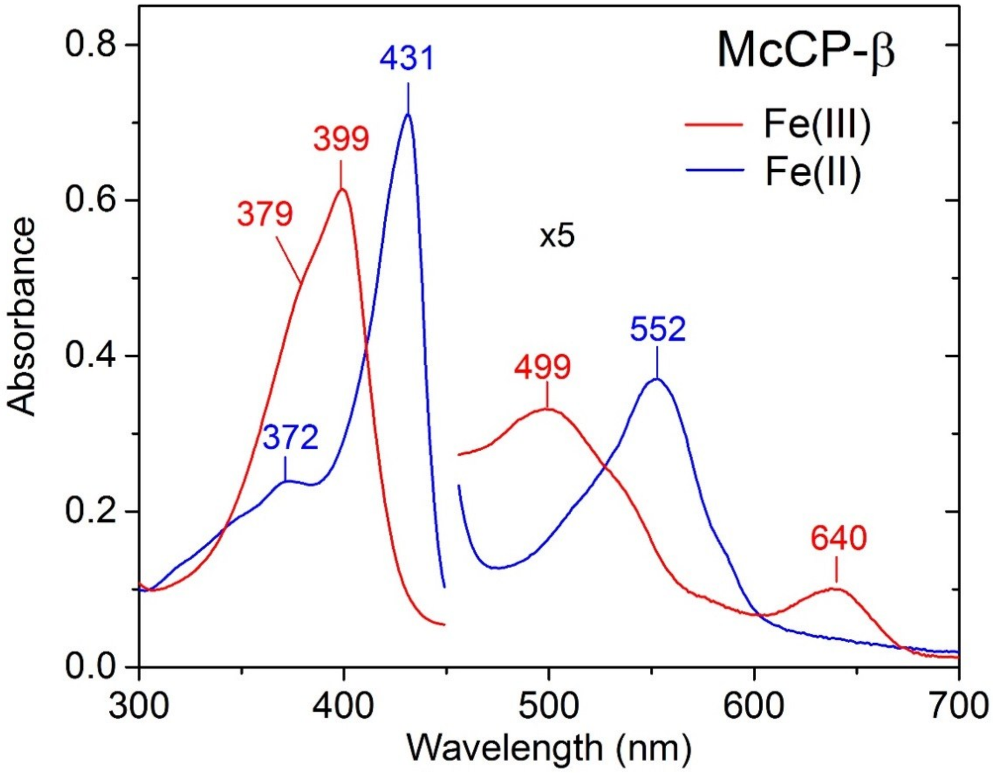

Figure S8. UV-visible absorbance spectra of McCP-β in the ferric and ferrous states.

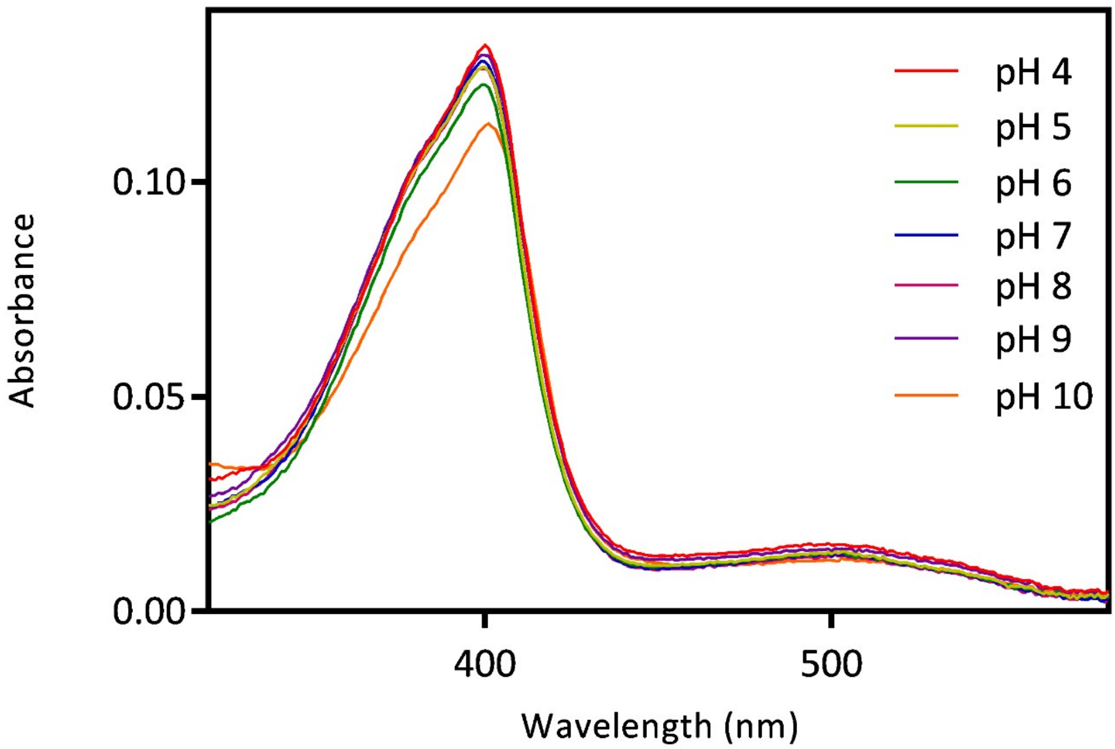

Figure S9: UV-Visible absorption spectra of ferric McCP-β at varying pH (4-10). Unlike cyts cp-α, changing the pH over this range does not change the absorbance features.

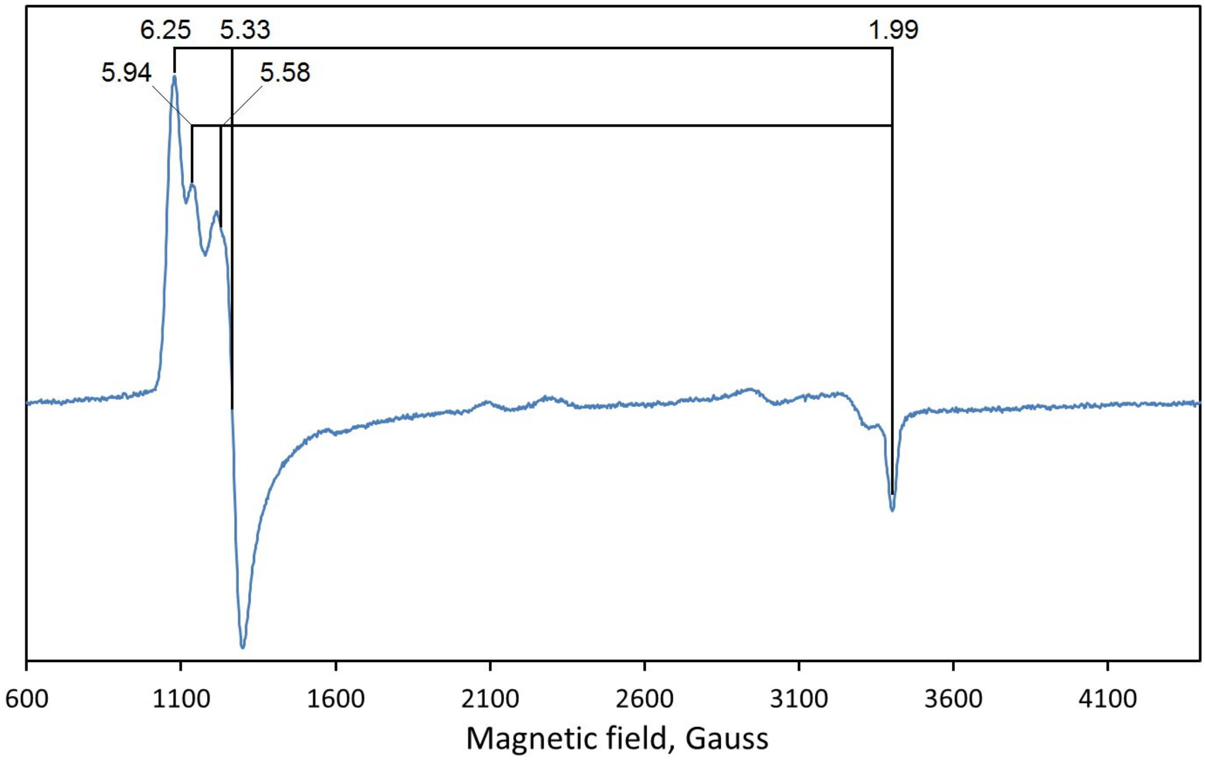

Figure S10. The EPR spectrum of 100 μM ferric McCP-β at pH 9. The major contribution to the EPR spectrum is from two high spin ferric forms – a major one with a higher rhombicity (g_1 = 6.25, g_2 = 5.33, g_3 = 1.99) and a minor, with a lower rhombicity – (g_1 = 5.94, g_2 = 5.58, g_3 = 1.99).

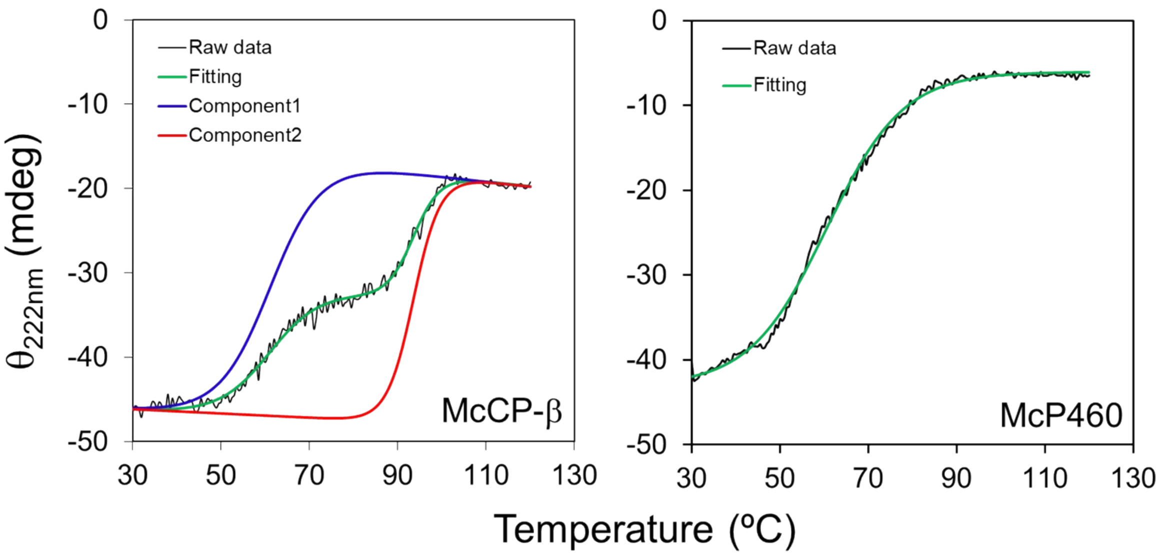

Figure S11: Thermal stability circular dichroism data for MCCP and McP460 with fits shown as green.

Protein degradation process in McP460

It was apparent during the purification of McP460 that, following the final round of size exclusion chromatography, the enzyme underwent a time-dependent spectroscopic shift similar to that reported previously for McP460 purified from the original organism [2]. In absorption spectra, the aged enzyme in the ferric state exhibited a shift in the Soret band from 419 nm to 415 nm with a visible colour shift from green to brown, figure S6. In the Fe(II) the characteristic 460 nm Soret band was shifted to 455nm and broadened in the aged enzyme. Zahn and co-workers [2] suggested that separation of McCP-β from McP460 during purification was an important factor in the observed spectral shift. Our observation that the shift occurs in recombinant McP460 (where McCP-β is absent) implies instead that this is an intrinsic property of McP460.

REFERENCES

1. J. A. Zahn, D. M. Arciero, A. B. Hooper and A. A. Dispirito, *Eur. J. Biochem.*, 1996, **240**, 684-691.
2. J. A. Zahn, C. Duncan and A. A. DiSpirito, *J Bacteriol*, 1994, **176**, 5879-5887.
3. D. J. Bergmann and A. B. Hooper, *Eur J Biochem*, 2003, **270**, 1935-1941.
4. J. D. Caranto, A. V. Vilbert and K. M. Lancaster, *Proc. Natl. Acad. Sci. USA*, 2015, **113**, 14704-14709.
5. M. A. Smith and K. M. Lancaster, *Biochemistry*, 2018, **57**, 334-343.
6. A. C. Vilbert, J. D. Caranto and K. M. Lancaster, *Chem. Sci.*, 2018, **9**, 368-379.
7. M. Numata, T. Saito, T. Yamazaki, Y. Fukumori and T. Yamanaka, *J Biochem*, 1990, **108**, 1016-1021.
